# Different N-Glycosylation Sites Reduce the Activity of Recombinant DSPAα2

**DOI:** 10.3390/cimb44090270

**Published:** 2022-08-31

**Authors:** Huakang Peng, Mengqi Wang, Nan Wang, Caifeng Yang, Wenfang Guo, Gangqiang Li, Sumei Huang, Di Wei, Dehu Liu

**Affiliations:** 1 Biotechnology Research Institute, Chinese Academy of Agricultural Sciences, Beijing 100081, China; 2 Biotechnology Research Institute, Guangxi Academy of Agricultural Sciences, Nanning 530007, China

**Keywords:** N-glycosylation site, plasminogen activator, fibrin sensitivity, DSPAα2, *Pichia* *pastoris*

## Abstract

Bat plasminogen activators α2 (DSPAα2) has extremely high medicinal value as a powerful natural thrombolytic protein. However, wild-type DSPAα2 has two N-glycosylation sites (N185 and N398) and its non-human classes of high-mannose-type N-glycans may cause immune responses in vivo. By mutating the N-glycosylation sites, we aimed to study the effect of its N-glycan chain on plasminogen activation, fibrin sensitivity, and to observe the physicochemical properties of DSPAα2. A logical structure design was performed in this study. Four single mutants and one double mutant were constructed and expressed in *Pichia pastoris*. When the N398 site was eliminated, the plasminogen activator in the mutants had their activities reduced to ~40%. When the N185 site was inactivated, there was a weak decrease in the plasminogen activation of its mutant, while the fibrin sensitivity significantly decreased by ~10-fold. Neither N-glycosylation nor deglycosylation mutations changed the pH resistance or heat resistance of DSPAα2. This study confirms that N-glycosylation affects the biochemical function of DSPAα2, which provides a reference for subsequent applications of DSPAα2.

## 1. Introduction

Thromboembolic diseases have become a significant global health risk due to the aging global population and drastic lifestyle changes. Despite many years of research, thrombolytic drugs often suffer from low specificity (causing non-specific bleeding) and a short half-life, raising further complications during the treatment of embolism patients [1,2,3]. The natural thrombolytic protein, vampire bat salivary plasminogen activator (DSPA), has exceptionally high fibrin sensitivity, it has shown a remarkable efficacy in animal models for thrombolysis, low incidences of systemic bleeding, low reocclusion rates, time windows that remain effective for up to nine hours, and the absence of potential neurotoxic effects. These superior properties make DSPA one of the best potential thrombosis treatments available [4]. DSPAα2 is a specific type of DSPA [5]. It is composed of four domains: a fibronectin type I domain (F), an epidermal growth factor region (EGF), a kringle 1 domain (K1), and a serine protease domain (P). The molecular structure of DSPAα2 is more similar to that of urokinase (u-PA), but the amino acid sequence is ~80% homologous to the tissue-type plasminogen activator (t-PA) [6].

The glycosylation modification of protein refers to the process of linking sugar chains to polypeptide chains via various enzymes, which happens before different degrees of folding and shifting take place to form glycosylated proteins. There are four types of glycan chain connections: N-glycosylation, O-glycosylation, C-glycosylation, and glycosylphosphatidylinositol (GPI)-mediated glycosylation [7]. The glycosylation of proteins is an essential post-translational modification that influences their physicochemical properties and functions. N-glycosylation can often increase the solubility of glycoproteins and also prevent their aggregation, thereby avoiding instances of degradation and reduction [8]. In terms of functionality, glycosylation modification can confer or change the unique biological activity of the related proteins, allowing for the more effective participation of modified proteins in various essential physiological functions [9]. Glycosylation modification is essential for pharmaceutical proteins. Specifically, the immunogenicity of N-glycan chains requires careful consideration. Furthermore, the glycosylation expressed by *Pichia* *pastoris* is different from that of mammalian glycosylation, which can be immunogenic in vivo and can also lead to accelerated clearance, thus limiting its therapeutic value [10]. The easiest solution to avoid the activation of immune responses is through deglycosylation mutation. However, mutating the glycosylation site will also change the protein’s immunogenic activity and affect the protein’s activity in vivo [11,12,13].

The glycosylation of modified proteins, such as recombinant t-PA (rt-PA), is also widely used in clinical therapy. DSPAα2 and t-PA share highly homologous N-glycosylation sites. However, the N-glycan chains of DSPAα2 expressed by *Pichia pastoris* are of the high-mannose type, while the t-PA N-glycan chains expressed by mammalian cells are of the hybrid type [10]. However, it is still unknown if DSPAα2’s activity is affected by N-glycosylation. To the best of our knowledge, there are no reports addressing whether the addition or a lack of N-glycosylation chains can affect the enzymatic activity of recombinant DSPAα2 (rDSPAα2). At present, DSPAα2 has been expressed using either the BHK system or *Pichia* *pastoris* expression system. The yield of the *Pichia* *pastoris* expression system has been slightly higher than that of the BHK system [14]. Therefore, this study constructed multiple glycosylation mutants of DSPAα2 based on glycosylation site prediction and homology modelling analysis and expressed them using the *Pichia pastoris* GS115 strain. We then aimed to compare the activity and stability of the constructed mutants. This paper elaborates on the effect that N-glycosylation has on the biochemical functions of DSPAα2 to provide a reference for the applications of DSPAα2.

## 2. Materials and Methods

### 2.1. Strains, Media, and Cultivation Conditions

*Escherichia coli* TOP10 (Zoman Bio, Beijing, China) was incubated at 37 °C in an LB medium (1% tryptone, 0.5% yeast extract, and 0.5% NaCl) containing kanamycin (50 μg/mL) for plasmid propagation. *P. pastoris* GS115 was used as a host for expression. The plasmid Ppic9k used was modified to switch the Aox promoter to the Gap promoter and was denoted as Ppic9k-Gap. The transformed *P. pastoris* was grown at 30 °C on an agar plate with a YPD medium (2% glucose, 2% peptone, and 1% yeast extract) containing 200 µg/mL of geneticin (Solarbio, Beijing, China). Additionally, the strain was then cultured in a BMGY medium (1% yeast extract, 2% peptone, 100 mM of potassium phosphate buffer, pH 6.0, 1.34% yeast nitrogen base without amino acids, 4 × 10^−5^% biotin, and 1% glycerol). Cultivation was performed at 30 °C under aerobic conditions with reciprocal shaking (200 rpm), and cell growth was monitored by measuring the optical density at 600 nm.

### 2.2. Construction of Strains Expressing Codon-Optimized DSPAα2

The oligonucleotide primers used in this study are listed in Supplementary Table S1. DSPAα2 was amplified by PCR with Phanta Max Super-Fidelity DNA Polymerase (Vazyme, Nanjing, China) using DSPAα2-9KY-F and DSPAα2His-9KY-R as primers and DSPAα2 (GenBank accession no. EF532598) as a template. The resulting PCR product also contained a 6*His-tag attached at the C terminus. The codon optimization of DSPAα2 was carried out by Sangon Biotech (Beijing, China). The product was digested with Xho I and Not I (NEB) and was cloned into the expression vector (Ppic9k-Gap under the Gap promoter), yielding Ppic9k-Gap-DSPAα2-His. The plasmid was then linearized by Sal I (NEB) and was used to transform *P. pastoris* GS115 via electroporation (400 Ω, 50 μF, 1.5 KV). The resulting strains were named rDSPAα2/GS115.

### 2.3. Construction of Strains Expressing Glycosylated Mutations of DSPAα2

N-glycosylation site analyses were carried out using the NetNGlyc 1.0 Server (http://www.cbs.dtu.dk/services/NetNGlyc/; accessed on 20 December 2021). We predicted that DSPAα2 had two N-glycosylation sites. Accordingly, the asparagine residues in the tripeptide sequences Asn-X-Ser/Thr (where X can be any amino acid except for proline) at sites N185 and N398 were targeted for mutagenesis. A homologous sequence alignment was performed using the Clustal Omega package (https://www.Ebi.ac.UK/Tools/MSA/clustalo/; accessed on 20 December 2021). The three-dimensional (3D) structure of DSPAα2 was fetched from the AlpahFold Protein Structure Database (https://alphafold.com/; accessed on 15 February 2022). We found two sites that could be mutated to add N-glycan chains. Finally, different residues were selected to produce four single mutants (QNGlyα2-1, QNGlyα2-2, ANGlyα2+1, and ANGlyα2+U), and one double mutant (QNGlyα2). Each target mutant plasmid was generated by site-directed mutagenesis using the plasmid Ppic9k-Gap-DSPAα*2*-His as a PCR template. The oligonucleotide primers used in this experiment are shown in Supplemental Table S1. The plasmid was linearized by Sal I (NEB) and was used to transform the *P. pastoris GS115* strain by means of electroporation (400 Ω, 50 μF, 1.5 KV). The resulting strains were named QNGlyα2/GS115, QNGlyα2-1/GS115, QNGlyα2-2/GS115, ANGlyα2+1/GS115, and ANGlyα2+U/GS115.

### 2.4. Purification of Proteins from Transformants

Each of the supernatant solutions containing the expressed proteins were collected by means of centrifugation at 4000× *g* for 5 min and were directly applied to the dialysis membranes (10 kDa). With respect to protein concentration, we covered the dialysis bag with PEG 20,000 solid dispersions. When the volume was concentrated to 10 mL, the solutions were purified using the Ni-NTA 6FF Sefinose™ Resin Kit (Sangon Biotech, Shanghai, China). Finally, the purified proteins were concentrated with the Amicon^®^ Pro System (Merck, Kenilworth, NJ, USA). The purity of these proteins was analyzed by the SDS-PAGE system.

### 2.5. Protein Concentrations

Protein concentrations were determined using the Bradford method and using a Bradford rapid protein quantification kit (Zoman Bio, Beijing, China) and 1 mg/mL of BSA as a protein standard [15].

### 2.6. SDS-PAGE Analysis

An SDS-PAGE analysis was carried out in 10% (*w*/*v*) polyacrylamide gel, and staining was conducted using Coomassie blue R-250 (Zoman Bio, Beijing, China) and a Pierce^TM^ Glycoprotein Staining Kit (Real-times Bio, Beijing, China). PNGase F (NEB, MA, USA) is the most effective enzyme for removing specific N-linked glycans (but not O-linked glycans) from glycoproteins. All protein samples were digested using PNGase and then compared with the undigested samples.

### 2.7. Fibrin Assay of DSPAa2 Enzyme Activity

Enzyme activity was detected by a modified fibrin assay as described in [16]. A fibrin agarose gel plate was prepared by mixing 15 mg of human fibrinogen in 5 mL of PBS (pH 7.4) with 20 mL of preheated 1% agarose solution in PBS and 10 U of thrombin in 2 μL of PBS. Purified solutions (~50 μL) were applied to each well on the plate and were incubated at 37 °C for 8 h. The activity of each sample was calculated by comparing the lytic area on the fibrin plate in contrast to the standard t-PA. The mean values were given as the average of three replicates (n = 3). Data are presented as means ± SD (n = 3). The significant differences between the means were evaluated using a *t*-test.

### 2.8. Blood Clot Lysis Activity Assay

An in vitro mice blood clot lysis activity assay was used as described by Prasad et al. [17]. Fresh blood was drawn from the retro-orbital sinuses of mice (*K*m, SPF). The blood (600 μL) was transferred to pre-weighed 1.5 mL Eppendorf tubes and incubated at 37 ℃ for 2 h. The serum was completely removed after clot formation. The tubes containing the clots were then weighed again. The clot weight was determined by subtracting the weight of each tube (clot weight = weight of clot-containing tube—empty tube weight). Then, 10 U of t-PA or 50 μL of DSPAα2 (of the proteins expressed in each mutation), dissolved in 1 M of PBS, was added to the tubes containing the clots. The treated samples were incubated at 37 °C overnight. The lysed fluid was completely absorbed from each tube using filter paper and the tubes were then re-weighed. The weight difference of each tube before and after incubation was calculated and the percentage of clot lysis in the treated and untreated samples was recorded. An average of three replicates (n = 3) was used, and the data were presented as the means ± SD (n = 3). The significant differences between the means were evaluated by a *t*-test.

### 2.9. Kinetics of S-2765 Hydrolysis

The assay volume was 0.15 mL and contained 10 nM samples, 100 μg/mL of fibrin/fibrinogen where stated (0.15 U/mL human thrombin in case of fibrin), and 0.01–5 mM of S-2765 in assay buffer (50 mM of Na_2_CO_3_, 0.1% Tween-80, pH 7.0). Individual assays were performed in triplicates and were repeated three times. The hydrolysis of S-2765 by thrombin was not detectable under these conditions. To correct for turbidity due to fibrin formation, the ΔA405-A490/min was monitored. Omitting the plasminogen activator, blanks were also performed in triplicates for every concentration of S-2765. As described above, the ΔA405/min was calculated and converted into [*pNA*]. According to the standard curve of [*pNA*], velocities were plotted against the S-2765 concentration and were analyzed using the Michaelis–Menten equation to obtain the kinetic parameters *K*m, *K*cat, and *K*cat/*K*m. Data were presented as means ± SD (n = 3).

### 2.10. Stability against Temperature and pH

The optimal reaction temperature of the wild-type and glycosylated mutations was analyzed after the enzymes were incubated with 1 M of PBS (pH 7.0) in the range of 30–90 °C for 1 h without substrate, and the activity was then measured by means of S-2765 hydrolysis. The optimal reaction pH was analyzed after the enzymes were incubated at 4 °C for 16 h with 1 M of PBS, pH 3.0–12.0, without substrate, and the activity was then measured in the same way as the means of the optimal reaction temperature. The mean values were given as an average of three replicates (n = 3) and the data were presented as means ± SD (n = 3). The thermostability was detected by a Nano-SPDF analysis, three replicates for each sample.

## 3. Results

### 3.1. Homology and Phylogenetic Analysis

**Figure 1:**
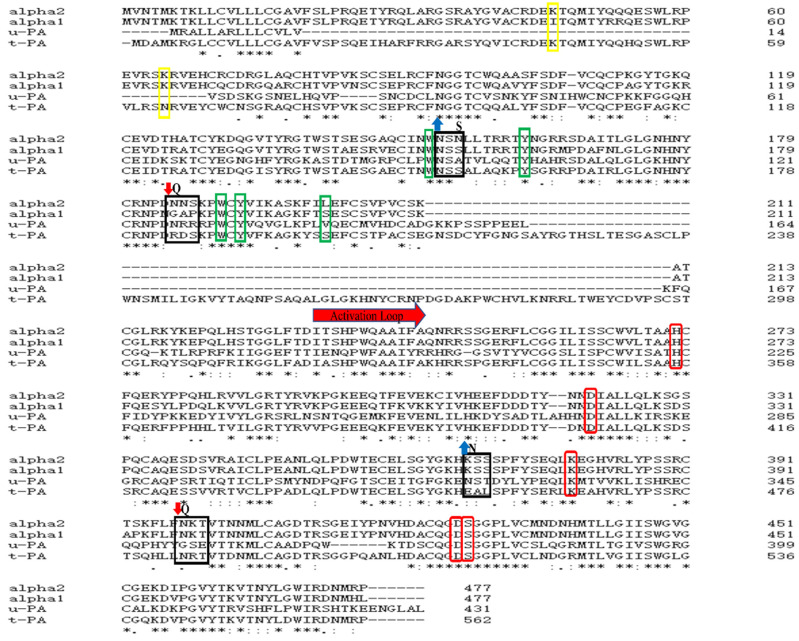
The Alignment of Animo acid sequence for DSPA α2, DSPA α1, t-PA and u-PA. Sequence alignment of DSPAα2 with DSPAα1, t-PA, and u-PA using the ClustalX program. The yellow rectangular boxes represent the sites (K46 and K65) associated with the fibrin-sensitive-related function in the F region. The green rectangular boxes represent the ligand-binding sites (W152, Y162, W190, Y192, and L201) in the EGF region. The red rectangular boxes represent the active pocket-associated sites (H272-D321-S428 and K379-D427) and the thick red arrow represents the activation loop. The black rectangular boxes indicate the glycosylation-related sites. The red arrows pointing down represent the deglycosylation-related mutation sites in DSPAα2, and the blue arrows pointing up represent the glycosylation-related mutation sites. The bold letters represent the mutated positions and the mutated amino acid residues.

The N-glycosylation predictions were performed using the NetNGlyc v1.0 Server (Figure S1). The amino acid sequence homology of DSPAα2, DSPAα1, t-PA, and u-PA were compared (Figure 1). Since these four proteins have highly homologous sequences, we speculated that the four proteins might also have structural and functional similarities. According to the results of the homologous comparison, four potentially mutable glycosylation sites (N153, N185, K368, and N398) were found (Figure 1, Supplemental Figure S2). DSPAα2 has 89.5% amino acid homology with DSPAα1, and they are highly similar in structure. Site N153 was derived from DSPAα1 and is highly homologous in structure to site N185, meaning that N-glycosylation mutations might enhance the sensitivity of DSPAα2 to fibrin and enhance the fibrinolytic activity. Therefore, we enabled site N153 to become a potential glycosylation site by mutating site N155 to S155. Site K368 is derived from u-PA. The N322 N-glycan chain in u-PA may slightly influence its activity; therefore, we mutated the homologous K368 site of N322 to N368 to verify its function [18]. Sites N185 and N398 were the wild-type N-glycosylation sites.

**Figure 2:**
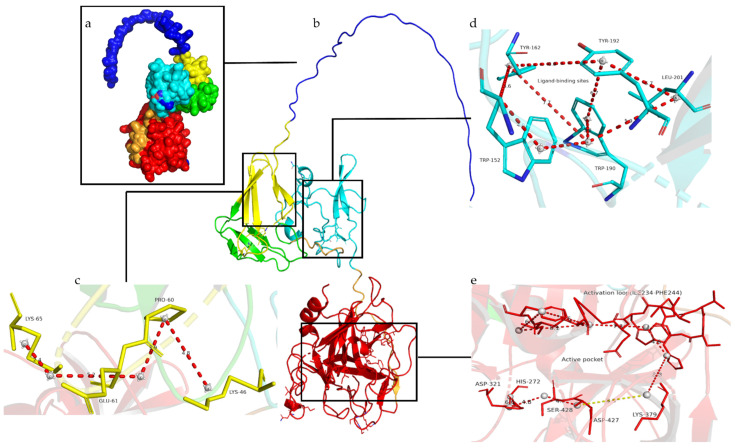
The Homology model of DSPAα2. (**a**) The solvent-accessible surface of DSPAα2. (**b**) The base ladders represent the internal structure of DSPAα2. The blue lines represent the signal peptide, yellow helices and sheets represent the F region, green represents the EGF region, cyan represents the K1 region, and red represents the P region. (**c**) A detailed view of the fibrin-sensitive-related sites in the F region. The yellow and blue sticks represent amino acids, and the red dotted lines represent the interaction distance between residues. (**d**) A detailed view of the ligand-binding sites in the K1 region. Amino acids are represented by cyan and blue sticks. The red dotted lines represent the interaction distance between residues. (**e**) A visual of the active pocket. Amino acids are represented by red sticks. The red dotted lines represent the interaction distance between residues, and the yellow dotted line represents the salt bridge connecting K379 and D427.

A homologous model can be used to predict the 3D structure of proteins, which would help us verify the feasibility of these mutations [19,20,21]. We retrieved the 3D structure of DSPAα2 from the AlphaFold Protein Structure Database and annotated the sites and domains that were closely related to its function (Figure 2) [22,23]. The fibrin sensitivity of DSPAα2 is mediated by fibrin binding, which involves the F domain, determinants within the K1 domain and P domain that have yet to be determined, and the absence of a plasmin-sensitive activation site [4]. The fibrin sensitivity of DSPAα2 was correlated with K46 and K65, which were in the F domain [4]. As shown in Figure 2c, K46 and K65 participate in fibrin recognition through the interaction of P60 and E61 to form a cross-like structure. The K1 region of DSPAα2 is highly homologous to the FN II type structure of fibronectin. The FN II structure of fibronectin mainly mediates the recognition and binding of its substrates, including fibrin. The ligand-binding sites in the FN type II structure are highly conserved in the K1 domain of DSPAα2 [24,25,26,27,28,29]. As shown in Figure 2d, W152, Y162, W190, Y192, and L201 are the ligand-binding sites. They are linked together by various molecular forces to form a rounded structure and participate in fibrin recognition. DSPAα2 has an active pocket composed of an active loop, consisting of Ile234-Phe244, three active sites (H272-D321-S428), and a salt bridge structure that consists of K379-D427. The active pocket enables DSPAα2 to exert high plasminogen activation activity in the absence of a plasmin-sensitive activation cleavage site [30]. As seen in the diagram (Figure 2e), the active loop mainly relies on the interaction force between the four benzene rings to maintain stability, while hydrogen bonds and hydrophobic forces connect the three active sites. The salt bridge connects the two separate structures to form the active pocket.

**Figure 3:**
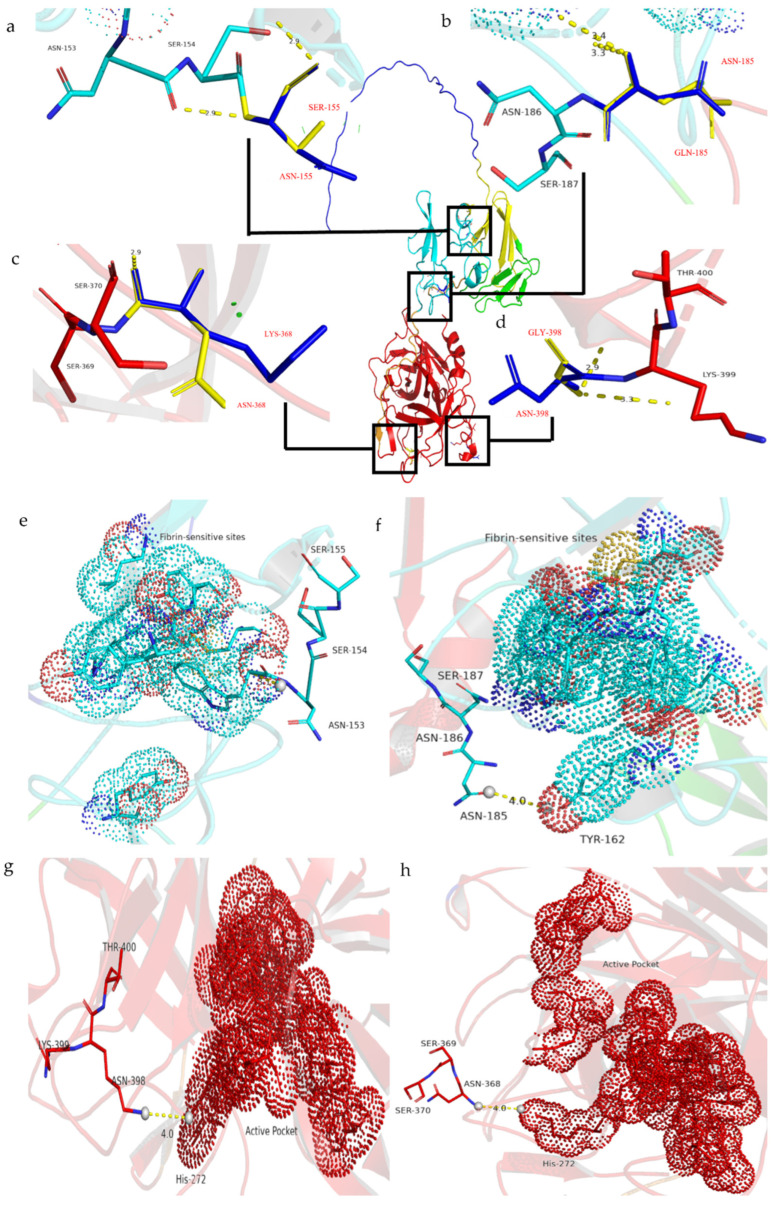
The residue interaction distance before and after mutation. (**a**–**d**) The blue sticks represent the original amino acid, the yellow sticks represent the mutated amino acid, and the yellow dotted line represents all of the polar distances of the sites: (**a**) N153 (N155S); (**b**) N185Q; (**c**) K368N; and (**d**) N398Q. (**e**–**h**) The sporadic dots represent the active surfaces of the sites, the blue and red dots indicate the ligand-binding sites, and the red dots denote the active pocket. The yellow dotted line represents the polar distance between the two linked atoms, and the sticks represent the selected glycosylation sites. Different colors indicate that they are in different domains: (**e**) N153(N155S); (**f**) N185Q; (**g**) K368N; and (**h**) N398Q.

In the spatial structure, all of the mutation sites are located near the key functional sites of DSPAα2. Sites N153 and N185 are located in the K1 domain close to the fibrin-sensitive sites and the ligand-binding sites, while sites K368 and N398 are located in the P domain close to the active pocket. We used the PyMOL tool to predict all of the interaction distances of the selected glycosylation sites, and we determined the corresponding functional sites. As can be seen from Figure 3a–d, none of the mutation sites changed their original polar distances. Therefore, when these sites were mutated, we believed that the functional or structural changes brought about by changes in the amino acid properties were not considered. To account for variability in protein structure and model accuracy, we defined two amino acid residues as those forming a contact where the distance between them was less than 10 Å [31,32,33,34,35]. The distances between sites N153 or N185 from the fibrin sensitive sites were more than 10 Å, so we considered that there might be no interaction between them (data not shown). It can be seen from Figure 3e–h that the distances between the four pairs of sites were all less than 10 Å (N153-W152, N185-W162, K368-K379, and N398-H272); therefore, there might be interactions between the sites. W152 and W162 belong to the ligand-binding sites involved in fibrin recognition, so we believed that the N-glycan chains attached to sites N153 or N185 could interact with the structure of the ligand-binding sites to interfere with fibrin recognition. K379 and H272 are the composition sites of the active pocket that exercise plasminogen activation activity, so we speculated that the N-glycan chains at the two sites might influence plasminogen activation activity. In summary, we assumed that the N-glycan chains on sites N153 and N185 affect the fibrin sensitivity of DSPAα2 by interacting with the ligand-binding sites, while sites N368 and N398 affect the serine protease activity of DSPAα2 by interfering with the function of the active pocket. The changes in the amino acid properties theoretically did not result in changes in the fibrin sensitivity or in the serine protease activity.

Ultimately, the experiment identified four mutation sites, and five mutants were constructed (Table 1).

### 3.2. Protein Level and Purification Using SDS-PAGE Analysis

**Figure 4:**
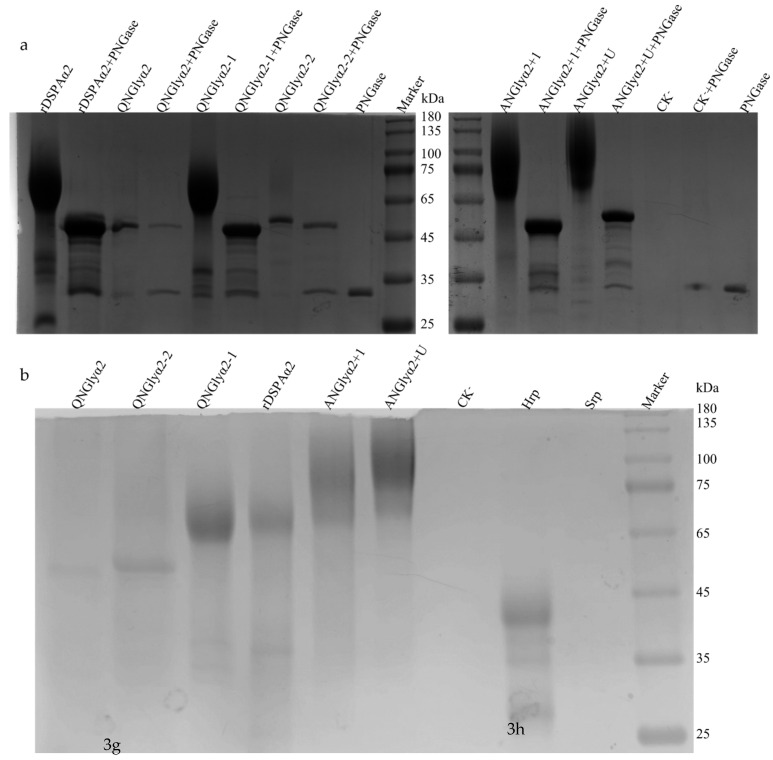
The SDS-PAGE analysis of the purified recombinant enzymes. (**a**) The coomassie blue staining. (**b**) The carbohydrate staining: Lane CK^-^, the cultivated supernatant of untransformed *Pichia pastoris*; Hrp, horseradish peroxidase; Srp: soy trypsin; and Marker, molecular mass markers purchased from Zoman Bio. The full-length gels are presented in Supplemental Figure S4.

To determine the enzymatic characteristics, rDSPAα2 and its mutants were expressed in *P. pastoris* and were purified using a Ni-NTA chromatography. All of the clones were identified by genomic PCR as well as RT-PCR analysis (Supplemental Figure S3). All of the purified samples were quantified by the Bradford method, and BSA was used as a protein standard to establish the standard curve. The final concentration of all samples was confirmed to be above 2 mg/L, the same as determined by Wei [14]. The effect of glycosylation on expression was also reported, but no principle was established [36,37,38]. The expression level of the wild-type rDSPAα2 was slightly higher than that of its N-glycosylation mutants, reaching 6629 IU/mg/L. This result is consistent with previous expression results of DSPAα1 in *P. pastoris* [39]. However, the relationship between the expression level and glycosylation of DSPAα2 was unclear (Supplemental Table S2). The proteins were identified by an SDS-PAGE analysis (Figure 4). It is known that glycosylation changes the protein size [40]. The SDS-PAGE analysis showed that QNGlyα2, which removed all N-glycosylation sites, was ~52 kDa in size, as predicted. QNGlyα2-2, which only retained the N185 site, was only slightly larger than QNGlyα2, at about 55 kDa. The size of rDSPAα2 was about 68 kDa, while the size of QNGlyα2-1 was slightly smaller than that of rDSPAα2. Based on the difference in molecular weight, we propose that the N-glycan chains on site N398 are more complex than the N-glycan chains on site N185. The molecular weights of ANGlyα2+1 and ANGlyα2+U, which were given additional glycosylation sites compared with the original protein, were both about 75 kDa, and ANGlyα2+1 was slightly smaller than ANGlyα2+U. After the deglycosylation of the N-linked glycans with PNGase F, the enzyme molecular mass decreased to ~52 kDa, which was consistent with the size of the N-deglycosylated mutant QNGlyα2 (Figure 4a). Moreover, carbohydrate staining results indicated that the N-glycan was successfully attached to each additional mutation as a single clear band (Figure 4b). The QNGlyα2 bands also appeared during glycoprotein staining as a result of the O-linked glycans, with several predicted O-glycosylation sites being found in rDSPAα2.

### 3.3. N-Glycosylation Mutation Reduced the Fibrinolytic Activity of DSPAα2

**Figure 5:**
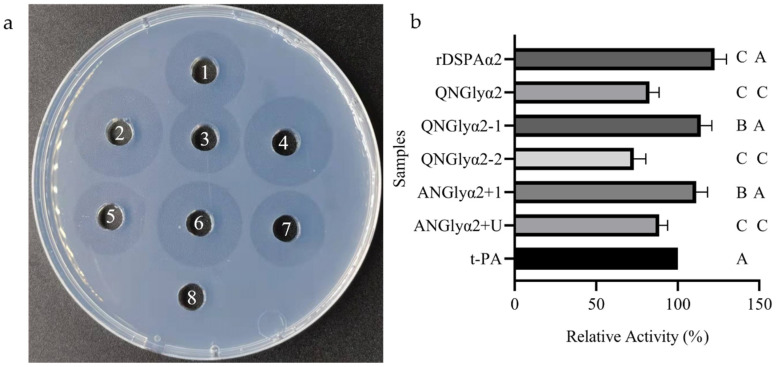
The fibrin assay and comparison of relative activity. (**a**) Results of the fibrin assay. Wells 1–8: t-PA, rDSPAα2, QNGlyα2, QNGlyα2-1, QNGlyα2-2, ANGlyα2+1, ANGlyα2+U, and control (PBS), respectively. (**b**) The relative activity of the rDSPAα2 and its mutants. The activity of all samples was compared to that of t-PA (10 U) to determine the relative activity, which is displayed in percentages. Values are mean ± SD of the three replicates. AB means 0.01 ≤ *p* ≤ 0.05, AC means *p* ≤ 0.01.

The fibrin assay is the most-used in vitro experimental method for determining plasminogen activation activity (Figure 5a). The standard t-PA (10 U) was used as a control and its coil diameter was defined as having 100% relative activity. Each sample was measured three times, and the average number was compared with the diameter of the standard t-PA to obtain the relative activity. The relative activity of rDSPAα2 was the highest out of all of the samples: ~50% higher than the lowest mutant, QNGlyα2. Furthermore, QNGlyα2-1 and ANGlyα2+1 were 10% lower than rDSPAα2 (Figure 5b). These results are consistent with those obtained in the structural prediction. The N153 and N185 sites are far away from the active pocket, and the N-glycan chain cannot participate in the construction of the sites related to plasminogen activation. The activity of the proteins with mutations at glycosylation sites (N398 and N368) associated with the active pocket was greatly reduced, indicating that the N-glycan chain at these sites was highly correlated with the plasminogen activation of DSPAα2. The introduction of site N368 did not increase activity as was expected. Experiments have shown that some glycosylation sites may introduce excessive glycosylation, which destroys or covers the structure of the enzyme’s active center, thereby reducing activity [41]. As the N368 site is close to the active pocket, the introduction of an additional N-glycosylation site may also lead to excessive glycosylation. Therefore, the coverage or destruction of the sites related to plasminogen activation subsequently inhibited activity.

**Figure 6:**
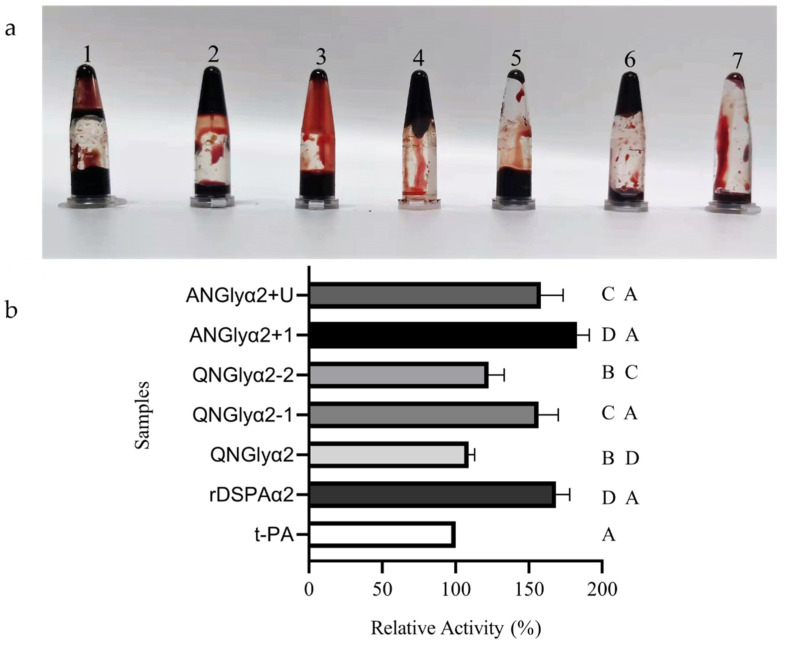
Comparison of clot lyses. (**a**) The results of the clot lysis assay. Tubes 1–7: rDSPAα2, QNGlyα2, QNGlyα2-1, QNGlyα2-2, ANGlyα2+1, ANGlyα2+U, and t-PA, respectively. (**b**) The relative activity of the rDSPAα2 and its mutants. The activity of all samples was compared to that of t-PA (10 U) to determine the relative activity, which is displayed in percentages. Values are mean ± SD of the three replicates. AB means 0.01 ≤ *p* ≤ 0.05, AC means 0.001 ≤ *p* ≤ 0.01, AD means *p* ≤ 0.001.

To simulate an in vivo thrombolytic environment (blood clots in blood vessels), the in vitro clot dissolution method was used (Figure 6a). The results were consistent with those obtained by the fibrin assay. The standard t-PA was used as a control, and the thrombolytic efficiency was defined as 100%. After removing the weight of the blood clot from each blood sample, ANGlyα2+1 was found to have the highest thrombolytic ratio, whereas QNGlyα2 had the lowest thrombolytic ratio (Figure 6b). The difference between these two ratios was over 80%. The relative activity of rDSPAα2 was only ~6% lower than that of ANGlyα2+1, which was a marked difference from the fibrin assay. However, from the perspective of the glycosylation sites, N-glycosylation at the N185 and N153 sites had little effect on plasminogen activation, while the N398 and N368 sites had a great influence on this activity.

In summary, the plasminogen activation of DSPAα2 was highly correlated with the N-glycosylation sites (N368 and N398) close to its active center. Glycosylation mutations around the active pocket greatly reduced plasminogen activation. The introduction of the glycosylation site belonging to uPA did not increase its plasminogen activation as had been expected. We speculated that the structure of its active center was coated or damaged by additional N-glycosylation. Therefore, when introducing this glycosylation site in the future, attention should be paid to its structural distance and relative position to the active pocket. The glycosylation mutation at the N185 and N153 sites had little effect on plasminogen activation.

### 3.4. N-Glycosylation Mutation Did Not Change the Physicochemical Properties of DSPAα2

**Figure 7:**
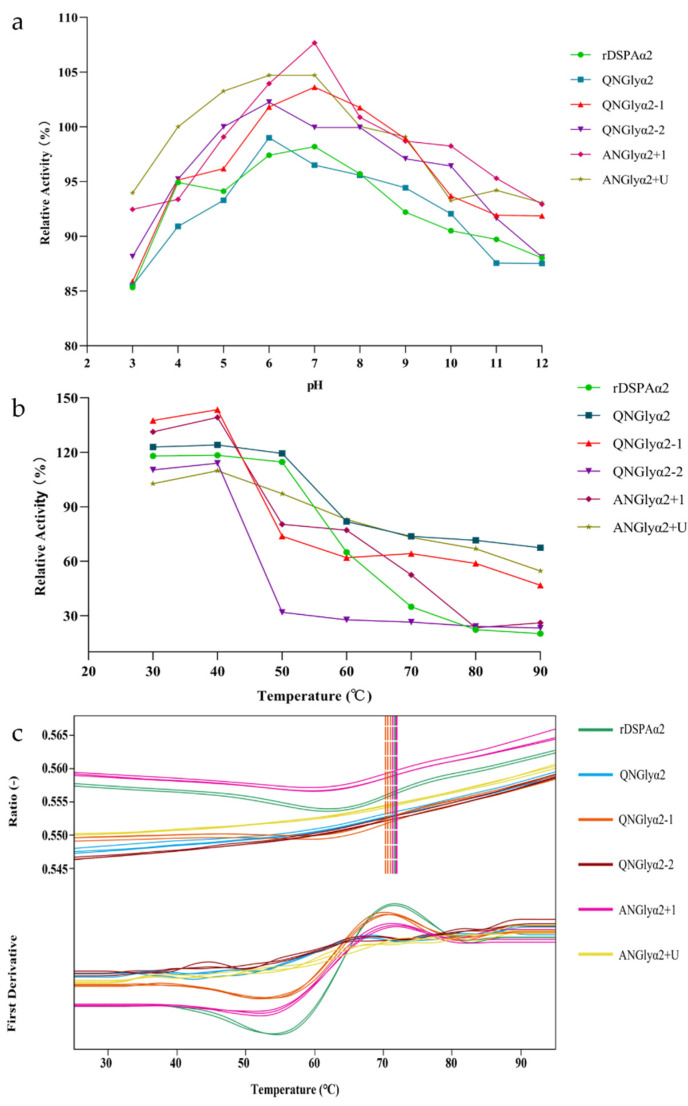
The physicochemical properties of rDSPAα2 and its mutants against pH and temperature. The curves of different colors represent the different protein samples: green represents rDSPAα2, blue represents QNGlyα2, orange represents QNGlyα2-1, purple represents QNGlyα2-2, pink represents ANGlyα2+1, and yellow represents ANGlyα2+U. (**a**) The optimal reaction pH. The activities of the different mutants at pH 7.0 were defined as 100%, and the activities at other pHs were compared with the activities at pH 7.0 to obtain their relative activities. The data were presented as the means ± SD (n = 3). (**b**) The optimal reaction temperature. The activities of the different mutants at 4 °C were defined as 100%, and the activities at other temperatures were compared with the activities at 4 °C to obtain their relative activities. The data were presented as the means ± SD (n = 3). (**c**) The thermostability. All samples were assayed using nano-SPDF, with three replicates for each sample.

Previous studies have shown that glycosylation affects protein stability [42]. As a potential pharmaceutical protein, DSPAα2 is active in blood at a pH of 7.0 and at temperatures around 36.9–37.9 °C. The optimal pH values displayed no obvious differences in activity, with a relatively high catalytic activity being observed against S-2765™ under neutral conditions (pH 6.0–7.0) (Figure 7a). The optimal temperature also displayed no significant differences in activity (Figure 7b). We measured six protein samples with a nanoDSF analysis. The F350nm/F330nm ratio in the six protein samples increased at 90 °C (Figure 7c). The variation trend of the scattering signals that were monitored during the healing process of the six samples tested was consistent. The scattering signals of all samples increased slightly at around 70 °C and increased sharply at around 90 ℃. This indicates that the aggregation of the six proteins began at 70 °C and occurred the most vigorously at 90 °C. We propose that the denatured unfolding of the sample began at 90 °C. Both the wild-type protein and the N-glycan mutants had the same physicochemical properties (optimal pH, temperature, and thermostability) relative to their working environment. Thus, most importantly, the mutations in the N-glycosylation sites did not alter the physicochemical properties of DSPAα2.

### 3.5. High Correlation between the N185 Site and Fibrinogen Specificity

Considering that the N153 and N185 sites may have affected the fibrin sensitivity of DSPAα2, S-2765 hydrolysis was used for identification. Mutating the two N-glycosylation sites led to changes in the glycan chains of this active recognition region, affecting the ability of DSPAα2 to recognize fibrin and thus decreasing plasminogen activation. Additional fibrin (fibrinogen) was added to the S-2765 hydrolysis as a supporting substrate to verify the fibrin sensitivity of DSPAα2 and its mutants.

The glycan chain at the N185 site is extremely important for the fibrin sensitivity of DSPAα2. Both rDSPAα2 and QNGlyα2-2 had high fibrin sensitivity. It can be seen from Table 2 that the *K*cat/*K*m ratios of the two proteins containing fibrin showed greater fold changes in comparison to those without any cofactors (~7–11 folds). The smaller the *K*m, the lower the substrate concentration required for the enzyme to carry out the reaction and the greater the affinity between the enzyme and substrate. The *K*m values of these two proteins also significantly decreased in the presence of fibrin compared with the absence of cofactors (~100-fold). We failed to observe this phenomenon in QNGlyα2 and QNGlyα2-1, which could be related to the loss of the N-glycan chain at the N185 site.

The N-glycan chain at the N398 site had a strong influence on the protease activity of DSPAα2. The *K*cat/*K*m of the three proteins (rDSPAα2, QNGlyα2-1, ANGlyα2+1) that were originally at the N398 site were much higher than those of QNGlyα2 and QNGlyα2-2 (at least 53 mM^−1^S^−1^ larger). *K*cat is the time it takes for an enzyme to convert a substrate. The larger the *K*cat value, the faster the enzyme can convert substrate molecules. The *K*cat values of the wild-type proteins (rDSPAα2, QNGlyα2-1 and ANGlyα2+1) can create about 30 folds of QNGlyα2 and QNGlyα2-2.

However, the introduction of the N-glycan chain at the N153 site caused ANGlyα2+1 to greatly reduce the fibrin sensitivity, and it was even unable to correctly recognize fibrinogen and fibrin. The *K*m/*K*cat ratio when fibrin was the cofactor was almost identical to the value when fibrinogen was the cofactor (Table 2). The N-glycan chain on site N153 is a very short distance from the ligand-binding sites (Figure 3e). As such, we suspected that the addition of the N-glycan chain caused the structure that was formed by the ligand-binding sites to be unable to accurately recognize fibrin.

In addition, fibrinogen also interacts with plasminogen, stimulating S-2765 lysis [43]. Therefore, the increase in activity that occurs when fibrinogen was used as a cofactor (Table 2) should not be considered as the effect of DSPAα2 on S-2765.

The kinetics of S-2765 hydrolysis were measured as described in the Materials and Methods Section. The kinetic parameters were derived from a nonlinear regression analysis of Michaelis–Menten plots depicting the velocity of [*pNA*] generation versus the S-2765 concentration. The stimulation factor observed in the presence of a fibrin/fibrinogen cofactor is presented in the second column from the right. We calculated the factor as the ratio of the bimolecular rate constants in the presence of fibrinogen or fibrin against those in their absence. The fibrin sensitivity in the right column was calculated as the ratio of activity (fibrin/fibrinogen). Values were the mean ± SD of the three replicates. The fitted Michaelis–Menten curves are displayed in Supplemental Figure S5.

## 4. Discussion

Over the past few years, many researchers have studied thrombolytic drugs with the intent to develop new ones. DSPAα2 has been found to be an efficient natural thrombolytic protein and is often compared with existing thrombolytic drugs due to its superior fibrin sensitivity and longer half-life. However, the focus of current studies has often on improving the expression efficiency, while the effect of N-glycosylation has not been studied. Experiments have shown that modifying the glycosylation of different expression systems is inconsistent, but this does not affect the DSPA activity [44]. Whether the loss or addition of N-glycan chains leads to changes in activity had not yet been studied [45]. In this study, four mutagenic glycosylation sites of DSPAα2 were discovered, and five glycosylation mutants were constructed by using homology alignment and homology modelling. The effect of N-glycans on DSPAα2 activity was verified by comparing the activity of different N-glycosylation mutants.

The N398 site is highly correlated with the plasminogen activation activity of DSPAα2. After mutating site N398 to Q398, the two related mutants (QNGlyα2, QNGlyα2-2) showed a large reduction in activity. Site N398 is structurally wrapped in the active pocket of DSPAα2 (Figure 2e). The N-glycan chain at this site may be involved in the construction of the active pocket. It is known that the spatial structure affects protein activity. Glycosylation plays an important role in the correct folding of proteins. Proteins without glycosylation may have reduced activity or even no activity [46,47]. The distance observed from the spatial structure is also an important factor to consider when determining whether the glycosylation sites affect activity. The longer the distance between the glycosylation site and the enzyme activity center (site), the smaller the probability of the glycosylation site affecting activity [48]. The N-glycan chain at the N185 site was involved in fibrin recognition. DSPAα2 is highly sensitive to fibrin, and its activity can be increased about 11 times when fibrin is involved in experiments. Based on our structural predictions, site N185 is located within the K1 region and close to the ligand-binding sites (W152, Y162, W190, Y192, and L201), where N-glycosylation modifications may affect the fibrin sensitivity of DSPAα2 (Figure 2d). When the N-glycan chain at this site was removed (QNGlyα2-1), its increase in activity (up to 2 folds) was greatly reduced compared with the wild-type.

Cell expression systems create expensive production costs. *Pichia pastoris*, a methylotropic yeast, has been developed as a host for heterologous protein expressions because of its low cost, high expression levels, efficient secretion, post-translation modification, proper protein folding, and potential for high-density cell fermentation [49]. However, some groups have reported that a few of the proteins expressed in yeast contain non-human classes of high-mannose-type N-glycans, which can be immunogenic in humans and can also lead to accelerated clearance, thus limiting their therapeutic value. As such, N-glycosylation is generally removed for medicinal proteins. This paper finds that this mutation is harmful to DSPAα2 activity. Therefore, the artificial N-glycan chain modification of DSPAα2 is necessary. The current N-glycosylation modification technology is quite mature. By using modification to humanize N-glycans, we can obtain highly active, recombinant rDSPAα2 that is more suitable for use in humans using the *Pichia pastoris* expression system [50,51]. This practice will result in new and efficient drugs for the treatment of thrombosis. Therefore, artificially modifying the N-glycan chains at the two sites (N185 and N398) should be considered to make it more suitable for humans and avoid immune reactions. We anticipate that scholars in related fields will join this area of research.

## 5. Conclusions

In this study, we found two N-glycosylation sites in DSPAα2 that may affect its activity using homology modelling, multiple sequence alignment, and specific mutants when using a *Pichia pastoris* expression system. For DSPAα2, N-glycosylation is critical for its plasminogen activation, especially at the N398 site. Its N-glycan chains are very close to the active pocket. The active pocket is vital for the plasminogen activation of DSPAα2. The sugar chains at this site either participate in the construction of the active center, function during plasminogen activation, or both. The N185 site is very close to the ligand-binding sites. These sites are key sites for fibrin-specific recognition. All of the mutants that had their N185 site removed exhibited low fibrin sensitivity. Moreover, the glycosylation sites introduced through homology comparison and modelling did not increase activity. We suspect that the reason for this was the proximity of these two sites (N153 and N368) to the critical active sites of DSPAα2. Through this study, we provide support for the expression strategy of DSPAα2 using *Pichia pastoris*.

## Figures and Tables

**Table 1:** N-glycosylation motifs of DSPAα2 and its mutants.

Enzyme	Motif	Modification	N-Glycosylation Sites	Mutation Sites
rDSPAα2	N153-N185-K368-N398	/	N185-N398	/
QNGlyα2	N153-Q185-K368-Q398	Deletion	/	N185Q-N398Q
QNGlyα2-1	N153-Q185-K368-N398	Deletion	N398	N185Q
QNGlyα2-2	N153-N185-K368-Q398	Deletion	N185	N398Q
ANGlyα2+1	N153-N185-K368-N398	Addition	N153-N185-N398	N155S
ANGlyα2+U	N153-N185-N368-N398	Addition	N185-N368-N398	K368N

N-glycosylation sites have only been represented by the site that the glycans linked to. Glycans linked to the Asn residues were modified in mutants. Mutation site indicates which site was directly mutated (Site N155 was mutated to make N153, a potential glycosylation site. For convenience, only site N153 is indicated in the paper).

**Table 2:** The kinetic parameters of the S-2765 hydrolysis by the DSPAs and t-PA in the presence or absence of a fibrin/fibrinogen cofactor.

Enzyme	Cofactor	*K*_cat_ ∗10^3^	*K*_m_ mM	*K*_cat_/*K*_m_ mM^−1^S^−1^	Stimulation Factor	Ratio Fbn/None
rDSPAα2	None	0.0955 ± 0.0085	0.9419 ± 0.0442	101.42 ± 5.52	1	
	Fbg	0.0947 ± 0.0021	0.4500 ± 0.0095	210.34 ± 4.86	2.1	
	Fbn	0.2043 ± 0.0040	0.0914 ± 0.0091	1141.79 ± 120.81	11.3	5.4
QNGlyα2	None	0.0023 ± 0.0009	1.1203 ± 0.0149	20.35 ± 0.60	1	
	Fbg	0.0021 ± 0.0006	1.0460 ± 0.0140	20.16 ± 0.53	0.9	
	Fbn	0.0030 ± 0.0009	0.5199 ± 0.0752	35.22 ± 4.12	1.7	1.9
QNGlyα2-1	None	0.0894 ± 0.0035	0.9567 ± 0.0283	93.40 ± 5.29	1	
	Fbg	0.0973 ± 0.0092	0.8782 ± 0.0440	110.78 ± 5.01	1.2	
	Fbn	0.1064 ± 0.0049	0.5638 ± 0.0128	188.84 ± 9.36	2.0	1.7
QNGlyα2-2	None	0.0028 ± 0.0002	1.5603 ± 0.0039	18.48 ± 6.67	1	
	Fbg	0.0037 ± 0.0023	0.7922 ± 0.0073	47.22 ± 6.92	2.6	
	Fbn	0.0046 ± 0.0002	0.3342 ± 0.0024	136.87 ± 11.30	7.4	2.8
ANGlyα2 + 1	None	0.1301 ± 0.0027	1.6877 ± 0.0080	77.37 ± 2.19	1	
	Fbg	0.0965 ± 0.0012	0.4300 ± 0.0036	224.44 ± 18.08	2.9	
	Fbn	0.0956 ± 0.0092	0.3970 ± 0.0034	240.36 ± 3.51	3.1	1.1

## Data Availability

The datasets generated and/or analyzed during the current study are available in the NCBI repository (OM630417, OM630418, OM630419, OM630420, OM630421). The other data supporting the conclusions of this article are included within the article and in the supplementary files.

## Supplementary Materials

The following supporting information can be downloaded at: https://www.mdpi.com/article/10.3390/cimb44090270/s1, Figure S1. The predicted N-glycosylation sites of DSPAα2; Figure S2. The alignment of nucleotide sequence for the DSPAα2 wild-type and mutants; Figure S3. The PCR and RT-PCR detection; Figure S4. The full-length gels; Figure S5. The fitted Michaelis–Menten curves; Table S1. The oligonucleotide primers used in this study; Table S2. The protein yields of rDSPAα2 and its mutants after purification.

## Abbreviations

DSPAα2: bat plasminogen activators α2; t-PA: tissue-type plasminogen activator; u-PA: urokinase; YPD: yeast peptone dextrose; BMGY: buffered glycerol-complex medium; LB: Luria–Bertani; BSA: bovine serum albumin; S-2765: a chromogenic substrate for the determination of Factor Xa; PBS: phosphate-buffered saline; *pNA*: a chromophore that can be cleaved from S2765 by specific enzymes so that free *pNA* can be detected by a spectrophotometer or microplate reader to determine the activity of the corresponding enzyme.
