# Supplementary material for: Different N-Glycosylation Sites Reduce the Activity of Recombinant DSPAα2
Source: Curr Issues Mol Biol. 2022 Aug 31;44(9):3930–47. doi: 10.3390/cimb44090270 (PMC9497888; doi:10.3390/cimb44090270)
Supplement: Supplementary file 1 [file cimb-44-00270-s001.zip › Figure S2. Alignment of nucleotide sequence for the DSPAα2 wildtype and mutants.pdf]

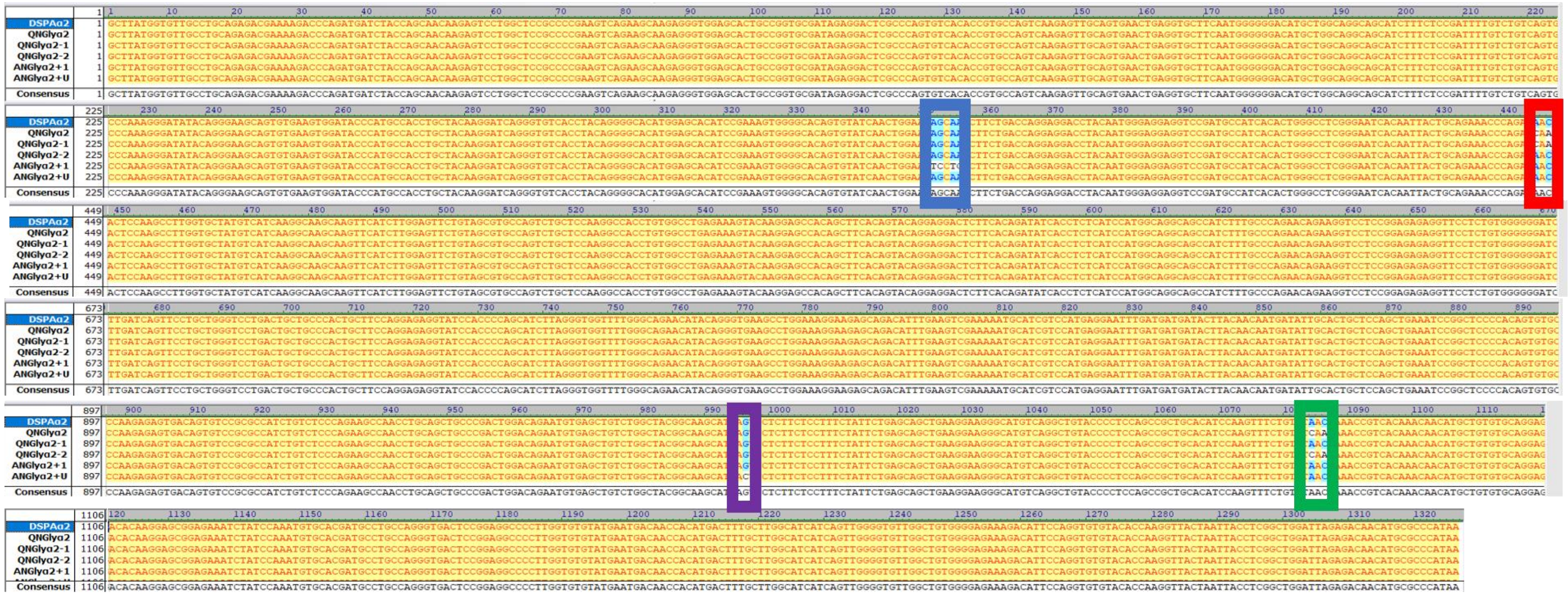

Figure S2. Alignment of nucleotide sequence for the DSPAα2 wildtype and mutants. The blue box identifies the nucleotide mutation sites of ANGlyα2+1; the red box identifies the nucleotide mutation sites of QNGlyα2 and QNGlyα2-1; the purple box identifies the nucleotide mutation sites of ANGlyα2+U; the greenlue box identifies the nucleotide mutation sites of QNGlyα2 and QNGlyα2-2.
