# Supplementary material for: Different N-Glycosylation Sites Reduce the Activity of Recombinant DSPAα2
Source: Curr Issues Mol Biol. 2022 Aug 31;44(9):3930–47. doi: 10.3390/cimb44090270 (PMC9497888; doi:10.3390/cimb44090270)
Supplement: Supplementary file 1 [file cimb-44-00270-s001.zip › Figure S3 PCR and RTPCR detection.pdf]

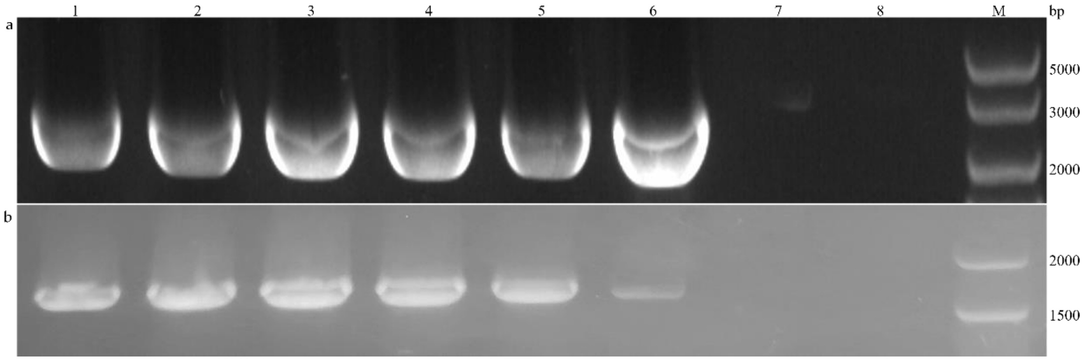

Figure S3. PCR and RTPCR detection. (**a**) PCR. (**b**) RT-PCR; lane 1-8: rDSPAα2, QNGlyα2, QNGlyα2-1, QNGlyα2-2, ANGlyα2+1, ANGlyα2+U, GS115, CK$^-$; M: Marker.
