# Supplementary material for: Different N-Glycosylation Sites Reduce the Activity of Recombinant DSPAα2
Source: Curr Issues Mol Biol. 2022 Aug 31;44(9):3930–47. doi: 10.3390/cimb44090270 (PMC9497888; doi:10.3390/cimb44090270)
Supplement: Supplementary file 1 [file cimb-44-00270-s001.zip › Figure S4 The full-length gels.pdf]

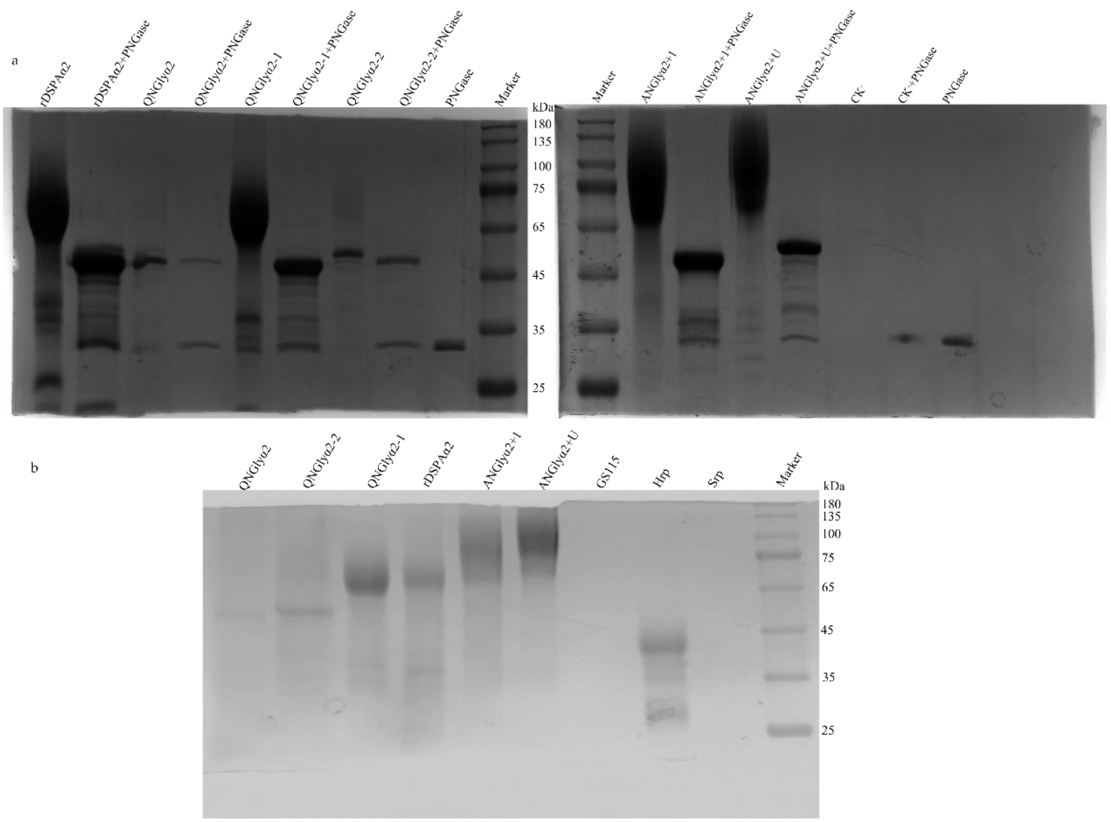

Figure S4. The full-length gels. (**a**) the full-length gels of Coomassie blue staining. (**b**) the full-length gels of Carbohydrate staining**.** Lane CK-, the cultivated supernatant of untransformed *Pichia pastoris*; Lane Hrp, *horseradish peroxidase*; Lane Srp: *soy trypsin*; Lane Marker, molecular mass markers purchased from Zoman Bio.
