# Supplementary material for: Different N-Glycosylation Sites Reduce the Activity of Recombinant DSPAα2
Source: Curr Issues Mol Biol. 2022 Aug 31;44(9):3930–47. doi: 10.3390/cimb44090270 (PMC9497888; doi:10.3390/cimb44090270)
Supplement: Supplementary file 1 [file cimb-44-00270-s001.zip › Table S1. The oligonucleotide primers used in this study.pdf]

Table S1. The oligonucleotide primers used in this study.

Primers	sequences
GAP-F	gacgcatgtcatgagattattgga
AOX-R	ggcaaatggcattctgacatcctc
ANGlyα2+U-F	tggctacggcaagcataactcctcttctcctttctattct
ANGlyα2+U-R	agaatagaaaggagaagaggagttatgcttgccgtagcca
ANGlyα2+1-F	acagtgtatcaactggaactcctcccttctgaccaggaggacctacaatg
ANGlyα2+1-R	tcctcctggtcagaagggaggagttccagttgatacactgtgccccactt
QNGlyα2-1-F	aacccagatcaaaactccaagccttggtgc
QNGlyα2-1-R	gcaccaaggcttggagttttgatctgggtt
QNGlyα2-2-F	tttctgtttcaaaaaaccgtcacaaacaac
QNGlyα2-2-R	gttgtttgtgacggttttttgaaacagaaa
DSPAα2-9KY-F	aaggaggagggtgtttccctcgagaaaagagcttatggtgttgcctgcagagacgaaaa
DSPAα2His-9KY-R	agtcatgtctaaggcgaaattcgcttagtggtgatggtgatggtgtgggcgcatgttgtc
