# Supplementary material for: Different N-Glycosylation Sites Reduce the Activity of Recombinant DSPAα2
Source: Curr Issues Mol Biol. 2022 Aug 31;44(9):3930–47. doi: 10.3390/cimb44090270 (PMC9497888; doi:10.3390/cimb44090270)
Supplement: Supplementary file 1 [file cimb-44-00270-s001.zip › Table S2. Protein yields of rDSPAα2 and its mutants after purification.pdf]

Table S2. Protein yields of rDSPAα2 and its mutants after purification.

Samples	Yields（IU in mg of protein /g of cell dry weight）
rDSPAaα2	6629
QNGlyα2	398
QNGlyα2-1	5021
QNGlyα2-2	119
ANGlyα2+1	2932
ANGlyα2+U	319
