# Supplementary figures and images for: Different N-Glycosylation Sites Reduce the Activity of Recombinant DSPAα2
Source: Curr Issues Mol Biol. 2022 Aug 31;44(9):3930–47. doi: 10.3390/cimb44090270 (PMC9497888; doi:10.3390/cimb44090270)

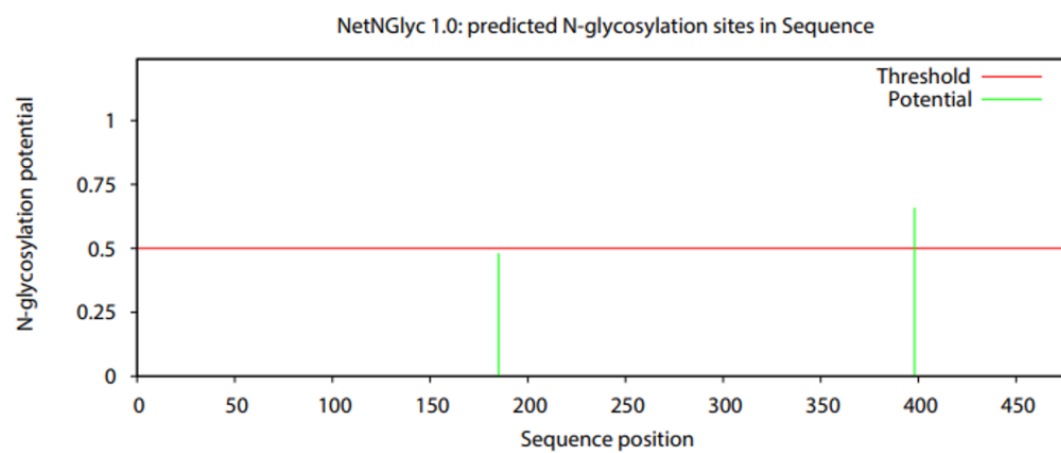

**Figure S1.** The predicted Nglycosylation sites of DSPA $\alpha$ 2. The predicted sites were N185 and N398.

Supplement: Supplementary file 1 [file cimb-44-00270-s001.zip › Figure S1. The predicted Nglycosylation sites of DSPAα2. .pdf]

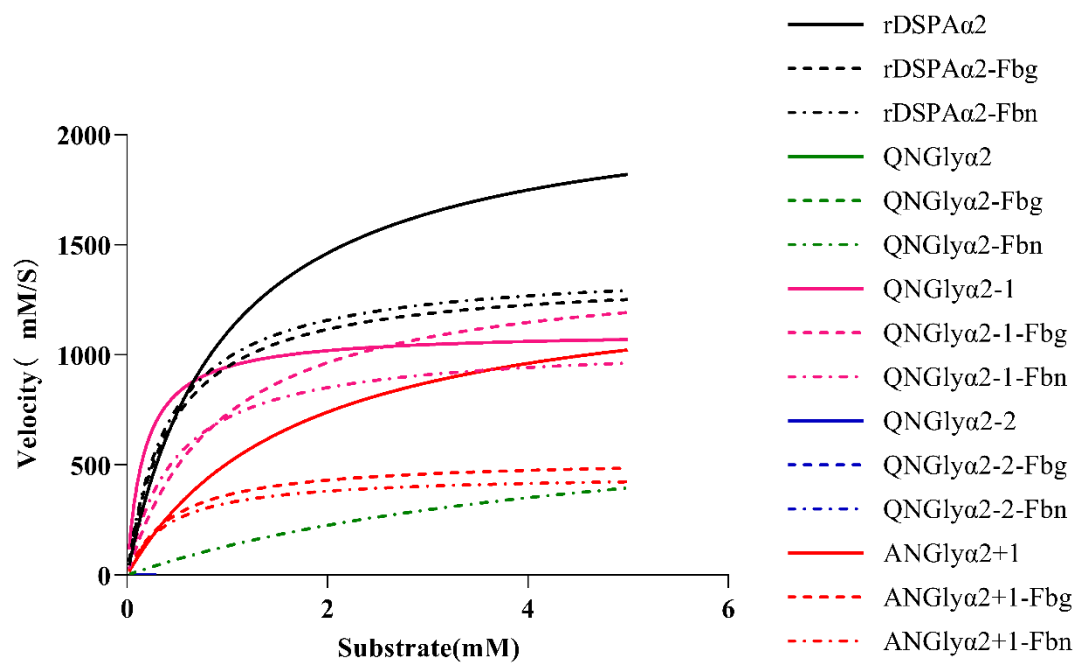

Figure S5. The fitted Michaelis-Menten curves.

Supplement: Supplementary file 1 [file cimb-44-00270-s001.zip › Figure S5. The fitted Michaelis-Menten curves.pdf]
